# ROS-Related miRNAs Regulate Immune Response and Chemoradiotherapy Sensitivity in Hepatocellular Carcinoma by Comprehensive Analysis and Experiment

**DOI:** 10.1155/2022/4713518

**Published:** 2022-05-09

**Authors:** Yangtao Xu, Xiaoqin He, Junjian Deng, Lin Xiong, Biao Chen, Jiayu Chen, Xiaoyu Zhang, Wenliang Chen, Xin Liu, Xinyao Hu, Jiayi Li, Shan Jiang, Yang Shen, Ximing Xu

**Affiliations:** ^1^Cancer Center, Renmin Hospital of Wuhan University, Wuhan, Hubei, China 430060; ^2^Pathology Department, Renmin Hospital of Wuhan University, Wuhan, Hubei, China 430060

## Abstract

Reactive oxygen species (ROS) plays an essential role in the development of cancer. Here, we chose ROS-related miRNAs for consensus clustering analysis and ROS score construction. We find that ROS is extremely associated with prognosis, tumor immune microenvironment (TIME), gene mutations, N6-methyladenosine (m6A) methylation, and chemotherapy sensitivity in hepatocellular carcinoma (HCC). Mechanistically, ROS may affect the prognosis of HCC patients in numerous ways. Moreover, miR-210-3p and miR-106a-5p significantly increased the ROS level and stagnated cell cycle at G2/M in HCC; the results were more obvious in cells after ionizing radiation (IR). Finally, the two miRNAs suppressed cell proliferation, migration, and invasion and promoted apoptosis in huh7 and smmc7721 cells. It indicated that ROS might affect the prognosis of HCC patients through immune response and increase the sensitivity of HCC patients to radiotherapy and chemotherapy.

## 1. Introduction

Hepatocellular carcinoma (HCC) is the sixth most frequently diagnosed cancer and the third leading cause of cancer death in the world in 2020 [1, 2]. The incidence of HCC is rising faster than other tumors, and the incidence of HCC in China is more than 10 times that of Europe and the United States, and its mortality rate ranks fifth among cancers [3]. There are many factors contributing to the high incidence of liver cancer, such as alcohol abuse, smoking, metabolic syndrome, and hepatitis virus. Especially in China, hepatitis B virus is the major risk factor for HCC [4]. Due to the insidiousness of the onset of HCC, patients are often diagnosed at an advanced stage. Although immunotherapy has developed rapidly in the past few years, improving the survival of patients with HCC, only a few patients with HCC could benefit from this treatment [5]. Therefore, novel molecular markers with more clinical utility are needed to improve diagnostic and prognostic prediction and guide the clinical treatment of HCC patients.

miRNAs are a group of approximately 21–25 nucleotides length and small endogenous single-stranded noncoding RNAs, which can negatively regulate gene expression via binding to the 3′-untranslated region of messenger RNA (mRNA) [6]. miRNA profiling can be used to predict cancer diagnosis and prognosis, for the pattern of miRNA expression can be correlated with cancer type, stage, and other clinical variables [7].

Recently, reactive oxygen species (ROS) have been shown to promote metastasis in a variety of cancers and play important roles in tissue homeostasis, cellular signaling, differentiation, and survival [8–13]. ROS are pleiotropic molecules or free radicals produced by numerous complex mechanisms. Its excessive production, failure of clearance mechanisms, and even insufficient antioxidants may lead to the accumulation of ROS, ultimately leading to oxidative stress [14]. Moreover, increasing evidence has suggested that numerous miRNAs have been linked to processes associated with ROS. Wang and colleagues find that significant increases in miR-34a-5p and miR-495-3p are consistent with ROS levels, but mir-34a-5p inhibition is reduced only in intestinal injury [15]. In addition, the research indicates that miR-15b inhibits ROS production by regulating SIRT4 [16]. However, studies of ROS-related miRNAs mainly focus on cardiac diseases, and the mechanism of hypoxia-induced miRNAs in tumors remains not clear.

In this research, we screened 36 ROS-related miRNAs from three reviews [14, 17, 18]. Then, 9 ROS-related miRNAs were selected and used for subsequent analysis. Through comprehensive analysis, ROS score was significantly corrected with prognosis, tumor immune microenvironment (TIME), gene mutations, m6A methylation, and chemotherapy sensitivity in HCC. In the experiments, miR-210-3p and miR-106a-5p extremely increased the ROS level and stagnated cell cycle at G2/M. Collectively, we confirmed the strong correlation between ROS and HCC through bioinformatics and experimental methods.

## 2. Materials and Methods

### 2.1. Dataset Source

The clinical data of HCC patients were downloaded from the University of California Santa Cruz (UCSC, https://xenabrowser.net/datapages/). The research included 374 tumor and 50 normal samples. The miRNA expression data of HCC patients were downloaded from the The Cancer Genome Atlas (TCGA) data portal by the “TCGAbiolinks” R package [19]. Furthermore, the mutation data of the TCGA-LIHC was downloaded from the websites (https://portal.gdc.cancer.gov/).

### 2.2. Identification of Consensus Clustering and Prognosis for ROS-Related miRNAs

According to the expression levels of 9 ROS-related miRNAs, HCC patients were clustered into different clusters by using R package “ConsensusClusterPlus” (http://www.bioconductor.org/).

### 2.3. The Calculation of ROS Scores

340 HCC patients were randomly divided into a validation dataset (170 patients) and training dataset (170 patients). Then, LASSO regression and univariate Cox analysis were used to identify five risk signatures, including miR-210-3p, miR-20b-5p, miR-144-5p, miR-106a-5p, and let-7a-5p. Then, the ROS scores were calculated by the formula: Ros Score = ∑_*i*=1_^*n*^Coefficient(miRNA*i*) × Expression(miRNA*i*).

### 2.4. Cell Culture

Human HCC huh7 and smmc7721 cell lines were purchased from Procell Life Science & Technology Co., Ltd. (Wuhan, China). The cells were cultured in high-glucose Dulbecco's modified Eagle's medium (DMEM) supplemented with 10% fetal bovine serum (FBS; Thermo Fisher Scientific, Inc., Waltham, MA, USA). All cells were cultured in a humidified incubator with 5% CO2 at 37°C.

### 2.5. Cell Transfection

The minics of miR-210-3p (5′CUGUGCGUGUGACAGCGGCUGA), miR-106a-5p (5′AAAAGUGCUUACAGUGCAGGUAG), and NC (5′UUCUCCGAACGUGUCACGUTT) were purchased from Suzhou GenePharma Co., Ltd. The Huh7 and Smmc7721 cells were seeded at 8 × 10^3^ cells/well in 96-well plates or 7.5 × 10^4^ cells/well in 6-well plates for 24 h, and then, the cells were transfected with MiR-210-3p/miR-106a-5p minics and NC minics for 48 h according to the Lipofectamine 3000 kit instructions.

### 2.6. Ionizing Radiation

The huh7 and smmc7721 cells were divided into normal and ionizing radiation groups. The ionizing radiation groups were exposed to 6 Gy X-ray.

### 2.7. qRT-PCR

qRT-PCR was performed with Hairpin-it™ Real-Time PCR miRNAs (GenePharma). The specific primers, miR-106a-5p and miR-210-3p, were purchased from Suzhou GenePharma Co., Ltd. Thermal cycles were as follows: 95°C for 3 min and 40 cycles of 95°C for 15 s, 62°C for 40 s. Melting curve analysis was used to confirm the specificity of amplification. The relative expressions of miRNAs to SNORD44 were determined using the comparative 2^-*ΔΔ*Ct^ method.

### 2.8. Measurement of ROS Production

Flow cytometry was used to assay ROS levels in Huh7 and Smmc7721 using a ROS assay kit (Shanghai Biyuntian Biological Co., Ltd.). Briefly, the cells were seeded in 6-well plates at a density of 2.5 × 10^5^ cells/well for 24 h. Next, the cells were incubated with 10 *μ*M of 2′,7′-dichlorodihydrofluorescein diacetate (DCFH-DA) for 15 min at 37°C in a dark room. A Beckman cytoFLEX flow cytometer was used to detect ROS levels. The data was analyzed by using the CytExpert 2.3 software.

### 2.9. Cell Cycle Analysis

Cell cycle analysis was conducted with a cell cycle staining kit (MultiScience Biotech Co., Ltd.). The transfected cells were seeded in 6-well plates at a density of 2.5 × 10^5^ cells/well for 72 h. Then, the cells were collected and incubated with 1 mL of DNA Staining solution and 10 *μ*M of permeabilization solution for 30 min in a dark room. A Beckman cytoFLEX flow cytometer was used to detect the cell cycle. The data was analyzed by using the CytExpert 2.3 software (Beckman Coulter, CA, USA).

### 2.10. Transwell Assay

Transwell assay was conducted to analyze the migrated and invasive abilities of cells. For cell migration, transfected SMMC-7721 and Huh7 cells (1 × 10^4^ cells/well) in 100 *μ*L serum-free DMEM medium were placed in the upper chambers (Costar, Corning, NY, USA), and 500 *μ*L DMEM medium containing 10% FBS was added to the lower chambers. Cells remaining on the top surface were removed with a cotton swab after the incubation for 24 h, while migrated cells through the membranes were fixed with 4% paraformaldehyde and stained with 0.1% crystal violet (Sigma, St. Louis, MO, USA). The stained cells were counted from five random fields under a microscope. For cell invasion, the experiment was performed following the same approach using transwell chambers pretreated with Matrigel (BD, San Jose, CA, USA).

### 2.11. Cell Proliferation Test

The transfected cells (7 × 10^3^) were seeded in 96-well plates; cell proliferation was assessed by the CCK8 reagent (Sigma-Aldrich) which was read from culture media at 450 nm (ELx800 Microplate Reader, BioTek Instruments, Winooski, VT, USA) at 0, 24, 48, and 72 h. Cell proliferation was expressed relative to the corresponding control.

### 2.12. Wound Healing Assay

The transfected cells were seeded in 6-well plates at a density of 2.5 × 10^5^ cells/well for 72 h, then draw a wound between the dense cells with a 200 *μ*L gun head. The gap closure was monitored under the microscope and a digital camera (CK30-SLP; Olympus, Tokyo, Japan) at 0, 24, and 48 h. Images were analyzed using the ImageJ version 1.52a software (National Institute of Health, Bethesda, MD, USA).

### 2.13. Cell Apoptosis Analysis

Apoptosis was assayed using the annexin V–phycoerythrin (PE)/7-aminoactinomycin D (7-AAD) or annexin V–adenomatous polyposis coli (APC)/7-AAD kit (Becton-Dickinson). The transfected cells were rinsed with ice-cold PBS and resuspended in 100 *μ*L of 1 × binding buffer. Then, the liquid was stained with 5 *μ*L 7-AAD and 5 *μ*L annexin V–APC/PE and incubated for 15 min in the dark. Then, another 400 *μ*L binding buffer was added into the mixture before cell apoptosis was detected on a Beckman cytoFLEX flow cytometer. The analysis of the above data was carried out using the CytExpert 2.3 software (Beckman Coulter, CA, USA).

### 2.14. Statistical Analysis

Statistical tests were carried out using GraphPad Prism 8.0 (GraphPad Software Inc., San Diego, CA, USA) and R version 4.0.2 (version 4.0.2, https://www.r-project.org/). “TCGAbiolinks,” “ConsensusClusterPlus,” “survival,” “glmnet,” “estimate,” “pRRophetic,” “maftools,” “edgeR,” and “timeROC” R packages were used.

## 3. Results

### 3.1. HBV-Related ROS miRNAs Were Significantly Associated with Clinical Features in HCC

**Figure 1:**
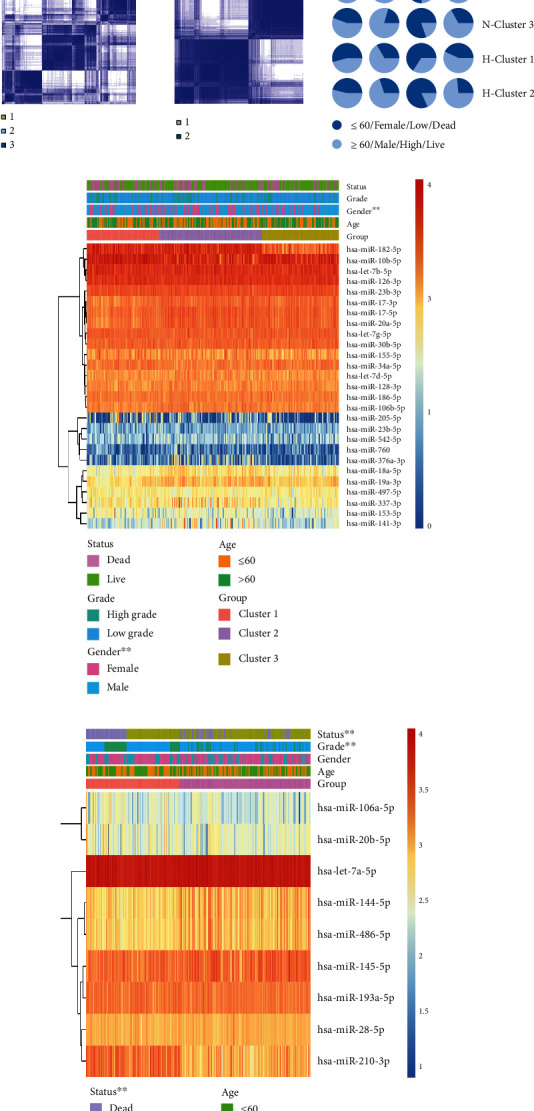
Consensus clustering for HBV- and none HBV-related ROS miRNAs with the clinical features and survival of HCC patients. (a) Expression levels of ROS-related miRNAs in HBV and normal samples. (b) Consensus clustering for HBV- and none HBV-related ROS miRNAs. (c) The proportion of clinical features in cluster types. (d) Clinical features in cluster1/2/3 for none HBV-related ROS miRNAs. (e) Clinical features in cluster1/2/3 for HBV-related ROS miRNAs. (f) Progonsis of cluster1/2/3. (g) Progonsis of cluster1/2. ^∗^*p* < 0.05, ^∗∗^*p* < 0.01, ^∗∗∗^*p* < 0.001, and ^∗∗∗∗^*p* < 0.0001.

Experimental evidences indicate that HBV X protein could cause DNA mutation through ROS generation [20]. To optimize the 36 ROS-related miRNAs, the difference of miRNAs between HBV patients and normal patients was further analyzed. As showed in Figure 1(a), 9 ROS miRNAs were significantly correlated with HBV, including miR-210-3p, miR-20b-5p, miR-144-5p, miR-106a-5p, miR-486-5p, miR-28-5p, miR-139a-5p, miR-145-5p, and let-7a-5p. Furthermore, according to the similarity between the expression level of m6A regulators and the proportion of fuzzy clustering measures, it is determined that *k* = 2 has the best clustering stability from *k* = 2 to 9, (Figure 1(b) and S1). HCC patients were clustered into cluster1 (*n* = 142) and cluster2 (*n* = 197), based on the expression levels of 9 HBV-related ROS miRNAs. Interestedly, we found that cluster1 was significantly associated with high grade, dead patients, and bad prognosis (Figures 1(e) and 1(g)). Also, HCC patients were clustered into cluster1 (*n* = 97), cluster2 (*n* = 138), and cluster3 (*n* = 104), according to the expression levels of 27 none HBV-related ROS miRNAs (Figure 1(b) and S2). However, the grade, status, and prognosis of HCC patients were not significantly different in cluster1/2/3 (Figures 1(c), 1(d), and 1(f)). The results showed that 9 HBV-related ROS miRNAs were more significant than to none HBV-related ROS miRNAs in HCC.

### 3.2. Correction between ROS Score and Clinical Features in HCC

**Figure 2:**
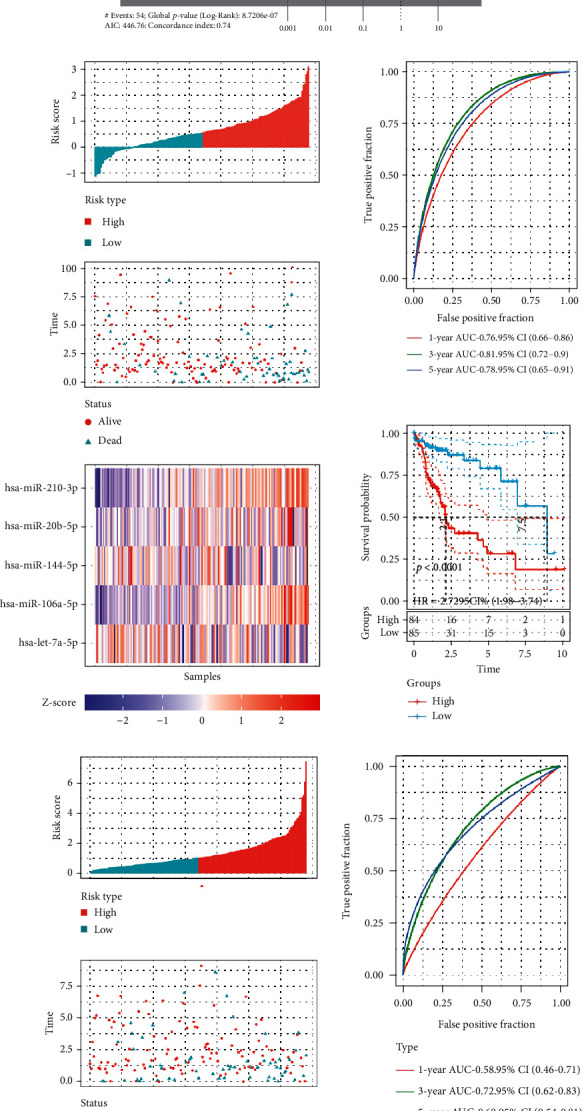
The construction of ROS score. (a) Five ROS-related miRNAs were selected through LASSO analysis. (b) The AUC of ROS score signature in training dataset. (c) The AUC of ROS score signature in validation dataset.

Firstly, 340 HCC patients were randomly divided into a validation dataset (170 patients) and training dataset (170 patients). Then, 5 candidate HBV-related ROS miRNAs were selected to calculate ROS score by using LASSO regression in the training dataset (Figure 2(a)). According to the expression of the candidate HBV-related ROS miRNAs, the formula went as follows: Ros Score = (2.3606 × let − 7a − 5p Expression) + (0.9226 × miR − 106a − 5p Expression) + (−0.3014 × miR − 144 − 5p Expression) + (−0.6911 × miR − 20b − 5p Expression) + (1.4139 × miR − 210 − 3p Expression). Furthermore, patients were divided into high- and low-risk groups, based on the median ROS score. The ROS score of high-risk group was higher, the low-risk group was instead. Interestedly, we found that high-risk groups were significantly associated with bad prognosis (Figure 2(b)). Moreover, the time-dependent receiver operating characteristic (ROC) curve was constructed. The time-dependent ROC curve (AUC) of five candidate HBV-related ROS miRNAs was 0.76, 0.81, and 0.78 at one year, three years, and five years (Figure 2(b)). With regard to the validation dataset, the AUC of the miRNAs was 0.58, 0.72, and 0.68 at one year, three years, and five years (Figure 2(c)). The results indicated that ROS score had a strong ability to predict prognosis in HCC.

**Figure 3:**
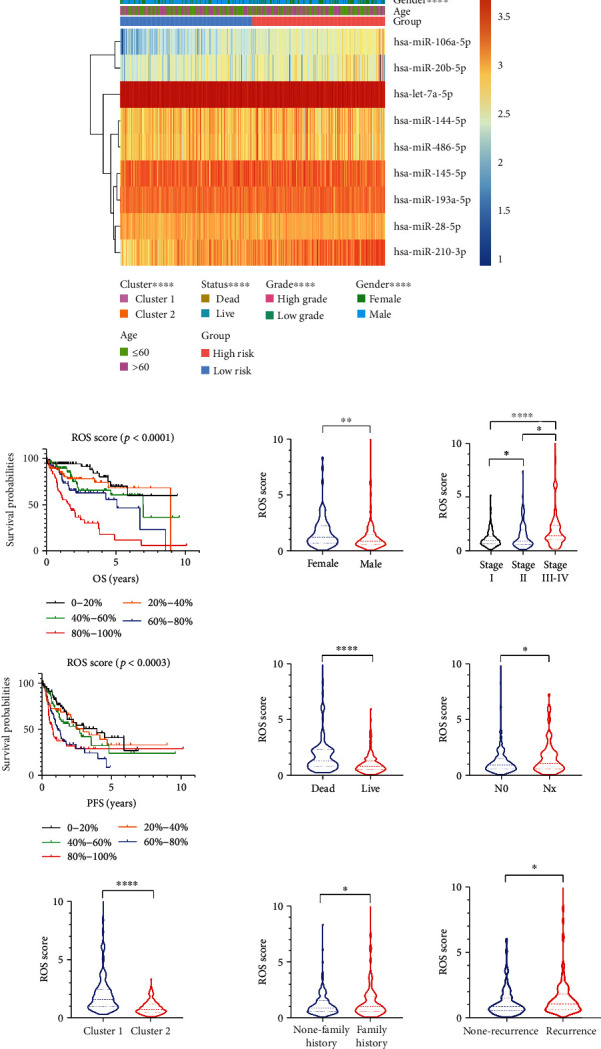
The correction between clinical features and ROS score. (a) Clinical features in the high- and low-risk groups. (b) ROS score in patients with different clinical characteristics. ^∗^*p* < 0.05, ^∗∗^*p* < 0.01, ^∗∗∗^*p* < 0.001, and ^∗∗∗∗^*p* < 0.0001.

Furthermore, clinical features between high- and low-risk groups were explored. We find that cluster1, high grade, dead status, female, and worse prognosis were significantly riched in high-risk group (Figure 3(a)). And the higher the ROS score, the more significant the correlation. Additionally, there were significant differences in tumor recurrence and lymph node metastasis between high- and low-risk groups (Figure 3(b)). The results showed that ROS score could effectively predict the clinical features of HCC patients.

**Figure 4:**
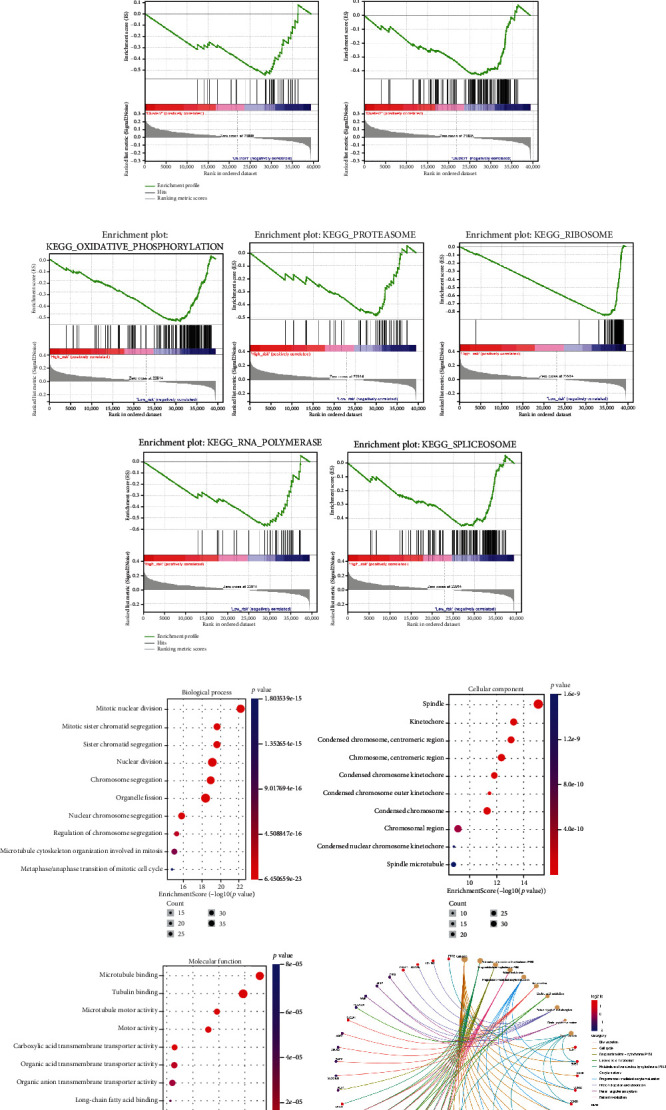
GSEA and GO enrichment analysis. (a) GSEA analysis in cluster1/2. (b) GSEA analysis for the high- and low-risk groups. (c) GO enrichment analysis for the common target genes of miR-210-3p and miR-106a-5p.

Additionally, GSEA was further analyzed in HCC. The pathways “oxidative_phosphorylation,” “proteasome,” “ribosome,” “polymerase,” and “spliceosome” were rich in cluster1 (Figure 4(a)). There were similar results in the high-risk group (Figure 4(b)). These pathways were directly or indirectly associated with cancers.

### 3.3. ROS Was Associated with TIME in HCC

**Figure 5:**
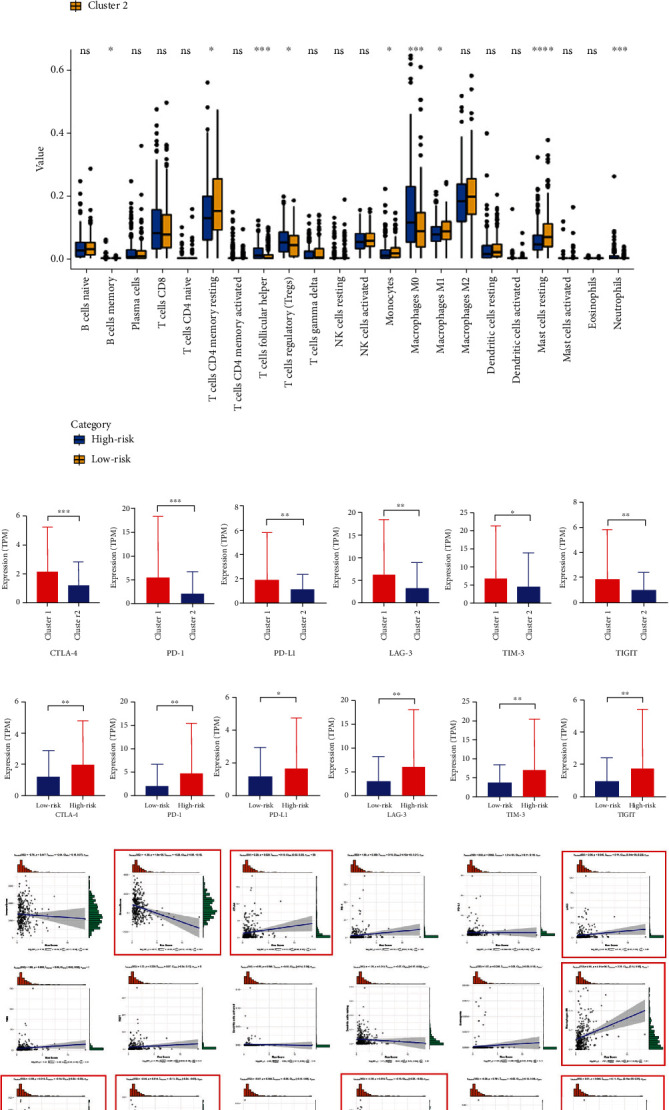
The correction between TIME and ROS score. (a, b) The expression of 22 immune cells in cluster1/2 and high/low-risk groups. (c, d) The expression of six immune markers in cluster1/2 and high/low-risk groups. (e) The correlation between TIME and ROS score. ^∗^*p* < 0.05, ^∗∗^*p* < 0.01, ^∗∗∗^*p* < 0.001, and ^∗∗∗∗^*p* < 0.0001.

Experimental evidences showed that the production of ROS in macrophages could affect both natural and acquired immunity and immune responses [21]. Therefore, the correction between ROS score and TIME in HCC was analyzed. We found that half of the immune cells (11/22) were significantly different in cluster1/2, especially “T cell CD4 memory resting,” “T cell follicular helper,” “Macrophages M0,” and “Macrophages M2” (Figure 5(a)). Moreover, 9 of the 22 immune cells were extremely different in the high- and low-risk groups, especially “T cells follicular helper,” “Macrophages M0,” “Mast cells resting,” and “Eosinophils” (Figure 5(b)). We further analyzed the effects of ROS on immune checkpoint inhibitors; six well-known immune markers were selected for analysis. We found that almost all markers (5/6) were distinct in cluster1/2 and high/low-risk groups (Figures 5(c) and 5(d)). All expression levels of six immune markers were higher in cluster1 and high-risk group, which were associated with worse prognosis in HCC patients. Furthermore, we analyzed the correction between immune cells/markers and ROS score. As showed in Figure 5(e), about one-third of the cells were significantly associated with ROS score. It indicated that ROS could affect the immune response and immunotherapy in HCC.

### 3.4. The Correction between ROS and Gene Muntion in HCC

**Figure 6:**
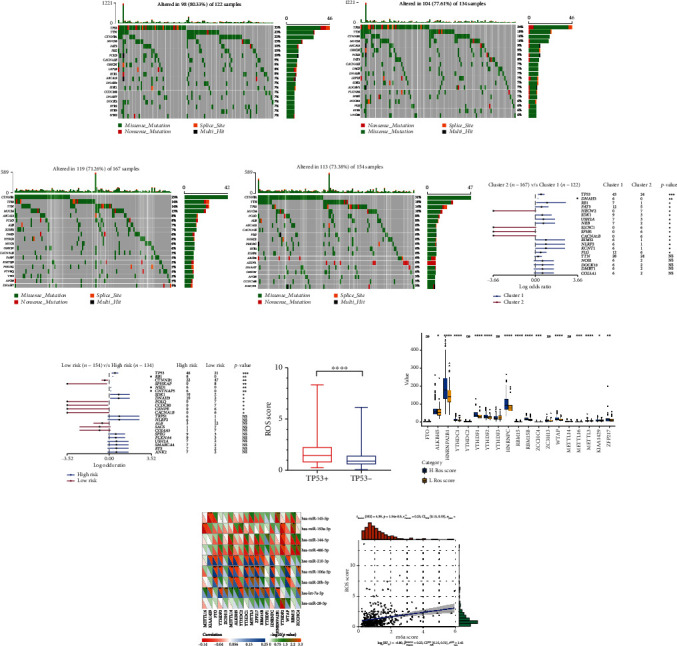
The correction between gene mutation, m6A methylation, and ROS score. (a) Gene altered of cluster1. (b) Gene altered of the high-risk group. (c) Gene altered of cluster2. (d) Gene altered of the low-risk group. (e, f) Gene mutations in cluster1/2 and high/low-risk groups. (g) The expression of ROS score in HCC patients with TP53+/-. (h) The expression of 19 m6A methylation regulators in high- and low-ROS score patients. (i) The correlation between 19 m6A methylation regulators and 9 ROS-related miRNAs. (j) The correlation between m6Ascore and ROS score. ^∗^*p* < 0.05, ^∗∗^*p* < 0.01, ^∗∗∗^*p* < 0.001, and ^∗∗∗∗^*p* < 0.0001.

ROS contributes to the accumulation of DNA mutations [22]. We further analyzed the effects of ROS on gene mutations in HCC patients. We found that the mutation rate of HCC patients was higher in cluster1 and high-risk group than in cluster2 and low-risk group (Figures 6(a)–6(d)). The highest mutation rate was TP53 in cluster1 and high-risk group (Figures 6(a) and 6(b)). Moreover, the mutated genes were more in cluster1 and high-risk group than in cluster2 and low-risk group (Figures 6(e) and 6(f)). As showed in Figure 5(g), ROS score of HCC patients with TP53+ was higher than patients with TP53- (Figure 6(g)). The results indicated that ROS could effect gene mutations, especially TP53 in HCC patients.

### 3.5. ROS Regulate m6A Methylation Level of HCC

Alteration of N6-methyladenosine (m6A) levels participates in cancer pathogenesis and progression [23–25]. We further analyzed the correction between ROS and m6A levels. The results showed that most of the m6A RNA methylation regulators (14/19) were more highly expressed in the high-ROS score group than in the low-ROS score group (Figure 6(h)). In addition, ROS-related miRNAs were significantly correlated with m6A RNA methylation regulators (Figure 6(i)). We further found that ROS score was positively correlated with the m6A score (Figure 6(j)). The results indicated that ROS could promote m6A methylation level of HCC.

### 3.6. ROS Induced Chemotherapy Sensitivity in HCC

**Figure 7:**
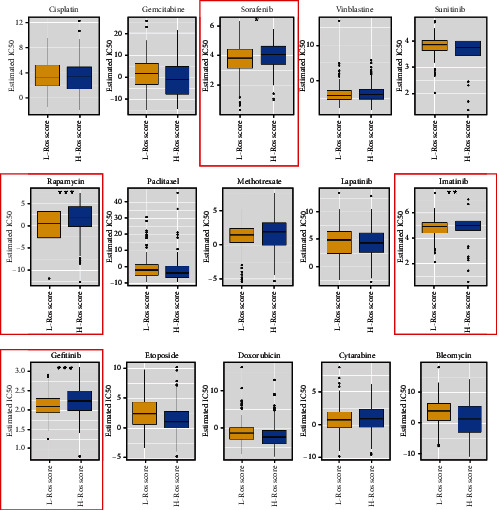
The correlation between chemotherapy agents and ROS score. ^∗^*p* < 0.05, ^∗∗^*p* < 0.01, ^∗∗∗^*p* < 0.001.

Many chemotherapeutic agents act on cancer through ROS production [26–28]. We further explored whether ROS could also affect the chemotherapy sensitivity in HCC. The correction between chemotherapy drug and ROS score was analyzed by using R package “pRRophetic”. As showed in Figure 7, “Sorafenib,” “Gefitinib,” “Rapamycin,” and “Lapatinib” were more highly expressed in high-ROS score patients. It showed that ROS could increase the chemotherapy sensitivity in HCC.

### 3.7. miR-210-3p and miR-106a-5p Were Associated with ROS and Cell Cycle

**Figure 8:**
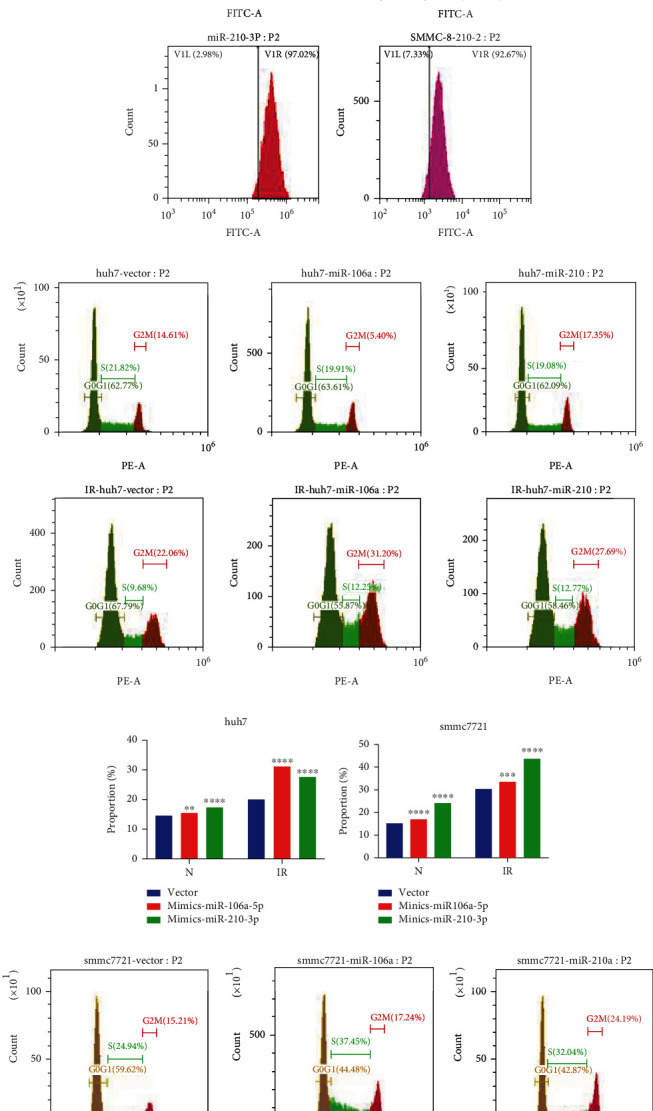
The ROS levels and cell cycle in HCC. (a) The ROS levels in huh7 and smmc7721 cells. (b) Cell cycle in huh7 cells. (c) The analysis of cell cycle in huh7 cells. (d) The analysis of cell cycle in smmc7721 cells. (e) Cell cycle in smmc7721 cells. IR: ionizing radiation; N: normal. ^∗^*p* < 0.05, ^∗∗^*p* < 0.01, ^∗∗∗^*p* < 0.001, and ^∗∗∗∗^*p* < 0.0001.

We further analyzed the overall survival (OS) of the five ROS-related miRNAs in HCC, only the OS of miR-210-3p and miR-106a-5p have statistical significance (Figure S3). We further find that miR-210-3p and miR-106a-5p increased ROS levels in huh7 and smcc7721 cells (Figure 8(a)). It is reported that ROS plays a role in radiation-induced cancer cell death [29]. Furthermore, we examined the effect of miRNAs on HCC cell cycle. Interestedly, miR-106a-5p and miR-210-3p stagnated the cell cycle at G2/M phases; the results were more obvious in cells after IR (Figures 8(b)–8(e)). It indicated that ROS-related miRNAs might improve the radiotherapy sensitivity of HCC. Additionally, GO enrichment analysis was performed for the common target genes of miR-210-3p and miR-106a-5p; the results were similar to GSEA analysis (Figure 4(c)).

### 3.8. miR-210-3p and miR-106a-5p Suppressed HCC Cells

**Figure 9:**
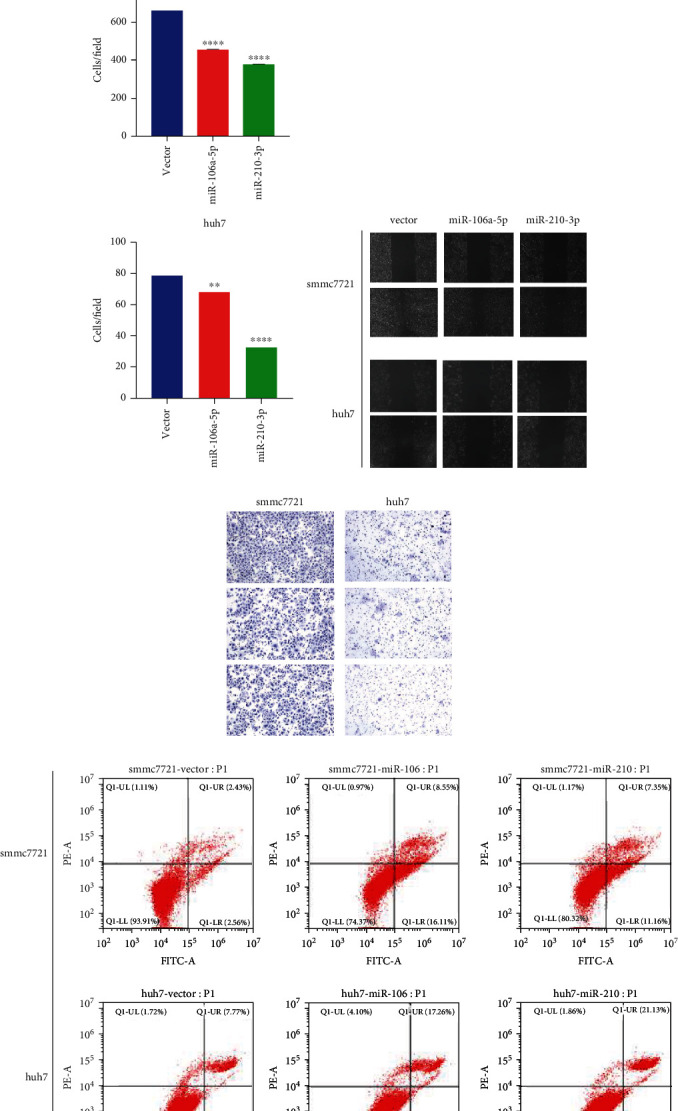
The biological function of miR-210-3p and miR-106a-5p in HCC. (a) The CCK8 value of miR-210-3p and miR-106a-5p in huh7 and smmc7721 cells. (b, d) miR-210-3p and miR-106a-5p inhibited huh7 and smmc7721 cell migration. (c, e) miR-210-3p and miR-106a-5p inhibited huh7 and smmc7721 cell invasion. (f, g) miR-210-3p and miR-106a-5p promoted apoptosis of huh7 and smmc721 cells. ^∗^*p* < 0.05, ^∗∗^*p* < 0.01, ^∗∗∗^*p* < 0.001, and ^∗∗∗∗^*p* < 0.0001.

We further analyzed the biological function of miR-210-3p and miR-106a-5p in HCC. We found that miR-210-3p and miR-106a-5p could suppress huh7 and smmc7721 cell proliferation (Figure 9(a)). Furthermore, two miRNAs inhibited cell migration and invasion (Figures 9(b)–9(e)). Additionally, two miRNAs promoted apoptosis of huh7 and smmc721 cells.

## 4. Discussion

The mechanisms of ROS in cancer are complex for numerous reasons [29]. First, ROS play a key role in the development of cancer [30–33]. Second, ROS induced G2/M arrest leads to affect the cell-cycle progression [34–37]. Third, chronic inflammation is regulated by ROS, which promote tumorigenesis [38, 39]. Forth, the expression levels of various tumor-related genes are regulated by ROS, including p53 [40–42]. Fifth, ROS are associated with cell apoptosis [43, 44]. Sixth, lots of chemotherapeutic and radiotherapeutic agents kill cancer cells by increasing ROS levels [45, 46]. Although ROS act on tumors through numerous pathways, the specific mechanisms remain unclear. ROS have a dual effect on tumors [29]. For example, ROS can promote tumor cell proliferation, such as bladder, liver, breast, lung, and ovarian cancer cells [47–50]. However, ROS also inhibits tumor cell proliferation, including liver, prostate, and breast cancer cells [51–53]. According to these previous reports, the role of ROS in tumors is contradictory and complex. Therefore, the mechanism of ROS in tumors needs to be further investigated. Currently, the effect of ROS in HCC needs further analyzed.

In this research, 36 ROS-related miRNAs were screened and divided into HBV-related ROS miRNAs (9) and none HBV-related ROS miRNAs (27). Then, HCC patients were divided into cluster1 and cluster2, based on the expression of the HBV-related ROS miRNAs. We found that cluster1 was significantly associated with high grade, dead patients, and bad prognosis. In addition, HCC patients were divided into cluster1, cluster2, and cluster3. However, the clinical features were not significantly different in cluster1/2/3. It indicated that HBV-related ROS miRNAs were more relevant to HCC patients. HBV can cause DNA mutation through ROS generation and is an important pathogenic factor of HCC [4, 20]. The miRNAs associated with both ROS and HBV are more relevant to HCC patients than mRNAs that are purely related to ROS. It was consistent with the above results. Additionally, HBV-related ROS miRNAs included miR-210-3p, miR-20b-5p, miR-144-5p, miR-106a-5p, miR-486-5p, miR-28-5p, miR-139a-5p, miR-145-5p, and let-7a-5p. Most of HBV-related miRNAs (6/9) were consistent with the previous reports [54–59].

Furthermore, 340 HCC patients were randomly divided into a validation dataset (170 patients) and training dataset (170 patients). Five candidate HBV-related ROS miRNAs were selected to calculate ROS score by using LASSO regression in the training dataset, including let-7a-5p, miR-106a-5p, miR-144-5p, miR-20b-5p, and miR-210-3p. ROS score was calculated by using the formula. Patients were divided into high-risk (high ROS score) and low-risk groups (low ROS score), based on the median ROS score. The high-risk groups were significantly associated with bad prognosis in the training dataset. Also, the similar result occurred in the validation dataset. Moreover, the AUC of five candidate HBV-related ROS miRNAs was 0.76, 0.81, and 0.78 at one year, three years, and five years in training dataset. In regard to the validation dataset, the AUC of the miRNAs was 0.58, 0.72, and 0.68 at one year, three years, and five years. AUC > 0.5 indicated prognostic ability, while AUC > 0.7 indicated a strong prognostic ability. The results showed that five candidate HBV-related ROS miRNAs had a strong prognostic ability.

Furthermore, we analyzed the relationship of TIME, mutation, m6A methylation, chemotherapy sensitivity, and ROS score in HCC. Interestedly, half of the 22 immune cells were significantly different in cluster1/2, especially “T cells CD4 memory resting,” “T cells follicular helper,” “Macrophages M0,” and “Macrophages M2” (*p* < 0.001). Moreover, ROS score was significantly corrected with immune checkpoint inhibitors; the expressions of CTLA-4, PD-1, LAG-3, TIM-3, and TIGIT were higher in the high-risk group. The results have also been verified in cluster1. Additionally, “StromalScore,” “CTLA-4,” “LAG-3,” “Macrophages M0,” “Macrophages M1,” “Macrophages M2,” “Neutrophils,” “Mast cells resting,” and “T cells regulatory (Tregs)” were extremely associated with ROS score (*p* < 0.05). These results showed that ROS could affect the immune response and immunotherapy in HCC. The increase of NADPH oxidase-derived extracellular and intracellular ROS is a major cause of oxidative stress, which contributes to functional changes in immune cells [60]. Interestedly, macrophages M0/1/2 were all associated with ROS score, and it indicated that ROS regulated tumor cells through macrophages. Moreover, ROS production in macrophages affects natural and acquired immunity and the immune response [21]. Thus, previous reports confirm our results.

In regard to gene mutation, the mutation rates of HCC patients with high ROS score were higher, and TP53 mutation rates were the highest among all genes in HCC patients with high ROS score. The results have also been verified in cluster1. Due to the lack of antioxidant damage repair system, mitochondria DNA (mtDNA) is more susceptible to ROS damage and mutation [61]. In turn, mtDNA mutations can increase ROS production, further aggravating the mutation effect and accumulating mutations, thus increasing the risk of tumor mutation [62]. What is more, ROS promote HCC cell survival by regulating TP53 degradation [63]. These researches confirm our results.

Regarding to m6A methylation, Zhuang and his colleagues find that m6A demethylase FTO could induce oxidative stress and ROS production and show impaired tumor growth [64]. Also, Yu and his colleagues find that ROS significantly induces global mRNA N6-methyladenosine (m6A) levels by modulating ALKBH5, to induce various biological processes quickly and effectively including DNA damage repair [65]. In this research, it is the first time to report that ROS is significantly associated with m6A in HCC. However, there are few reports about ROS and m6A; the mechanism of ROS and m6A needs further exploration.

Many chemotherapeutic agents kill tumor cells through ROS production [29]. For example, procarbazine is one of the first drugs to be developed based on its ROS-producing properties [45]. Until to now, the drug is approved to treat Hodgkin's lymphoma, non-Hodgkin's lymphoma, and primary brain tumors [66, 67]. In our research, we analyzed the difference of chemotherapy drugs between high-ROS score patients and low-ROS score patients. The results showed that “Sorafenib,” “Gefitinib,” “Rapamycin,” and “Lapatinib” were more highly expressed in high-ROS score patients, which indicated that ROS could increase the chemotherapy sensitivity in HCC.

Finally, our experiments showed that ROS-related miRNAs miR-210-3p and miR-106a-5p could increase the ROS level of HCC. Moreover, these two miRNAs could stagnate HCC cell cycle at G2/M; the results were more obvious in cells after IR. The relative radiosensitivity of cells is determined by the cell cycle stage. Cells are most sensitive to radiation in G2/M phase, less sensitive in G1 phase, and least sensitive in late S phase [68].

Therefore, ROS-related miRNAs miR-210-3p and miR-106a-5p can increase the radiotherapy sensitivity of HCC cells. ROS play a role in radiation-induced cancer cell death, including lung cancer, prostate cancer, and breast cancer [69–71]. Our experimental results provide data support for the role of ROS in HCC radiotherapy. Moreover, the experimental results showed that miR-210-3p and miR-106a-5p suppressed HCC cell proliferation, migration, and invasion. And two miRNAs promoted apoptosis of huh7 and smmc721 cells. It indicated that miR-210-3p and miR-106a-5p played a key role in the biological function of HCC.

In summary, we systematically assessed the relationship of clinical futures, TIME, muntion, m6A methylation, chemotherapy sensitivity, and ROS score in HCC. HCC patients were divided into cluster1/2 and high/low-risk groups, based on the expression of ROS-related miRNAs and ROS score. High ROS score was significantly with worse prognosis, immune cells, and immune checkpoint inhibitors. High ROS score also regulated gene mutation, m6A methylation level, and increased chemotherapy sensitivity in HCC. These results were confirmed in both cluster1/2 and high/low-risk groups. Moreover, ROS-related miRNAs miR-210-3p and miR-106a-5p significantly increased the ROS level and radiotherapy sensitivity and played a key role in the biological function of HCC. Therefore, ROS might improve the radiotherapy sensitivity of HCC patients, which could provide a new treatment strategy for HCC patients.

## Data Availability

The clinical data of HCC patients were downloaded from the University of California Santa Cruz (UCSC, https://xenabrowser.net/datapages/). The research included 374 tumor and 50 normal samples. The miRNA expression data of HCC patients were downloaded from the The Cancer Genome Atlas (TCGA) data portal by the “TCGAbiolinks” R package. Furthermore, the mutation data of the TCGA-LIHC was downloaded from the websites (https://portal.gdc.cancer.gov/).

## Supplementary Materials

Supplementary Materials Figure S1: consensus clustering matrix from *k* = 2 to 9 for 9 HBV-related ROS miRNAs. Figure S2: consensus clustering matrix from *k* = 2 to 9 for 27 none HBV-related ROS miRNAs. Figure S3: the OS of miR-210-3p and miR-106a-5p in HCC.
