# Supplementary material for: ROS-Related miRNAs Regulate Immune Response and Chemoradiotherapy Sensitivity in Hepatocellular Carcinoma by Comprehensive Analysis and Experiment
Source: Oxid Med Cell Longev. 2022 May 9;2022:4713518. doi: 10.1155/2022/4713518 (PMC9110211; doi:10.1155/2022/4713518)
Supplement: Supplementary Materials — Figure S1: consensus clustering matrix from k = 2 to 9 for 9 HBV-related ROS miRNAs. Figure S2: consensus clustering matrix from k = 2 to 9 for 27 none HBV-related ROS miRNAs. Figure S3: the OS of miR-210-3p and miR-106a-5p in HCC. [file 4713518.f1.docx]

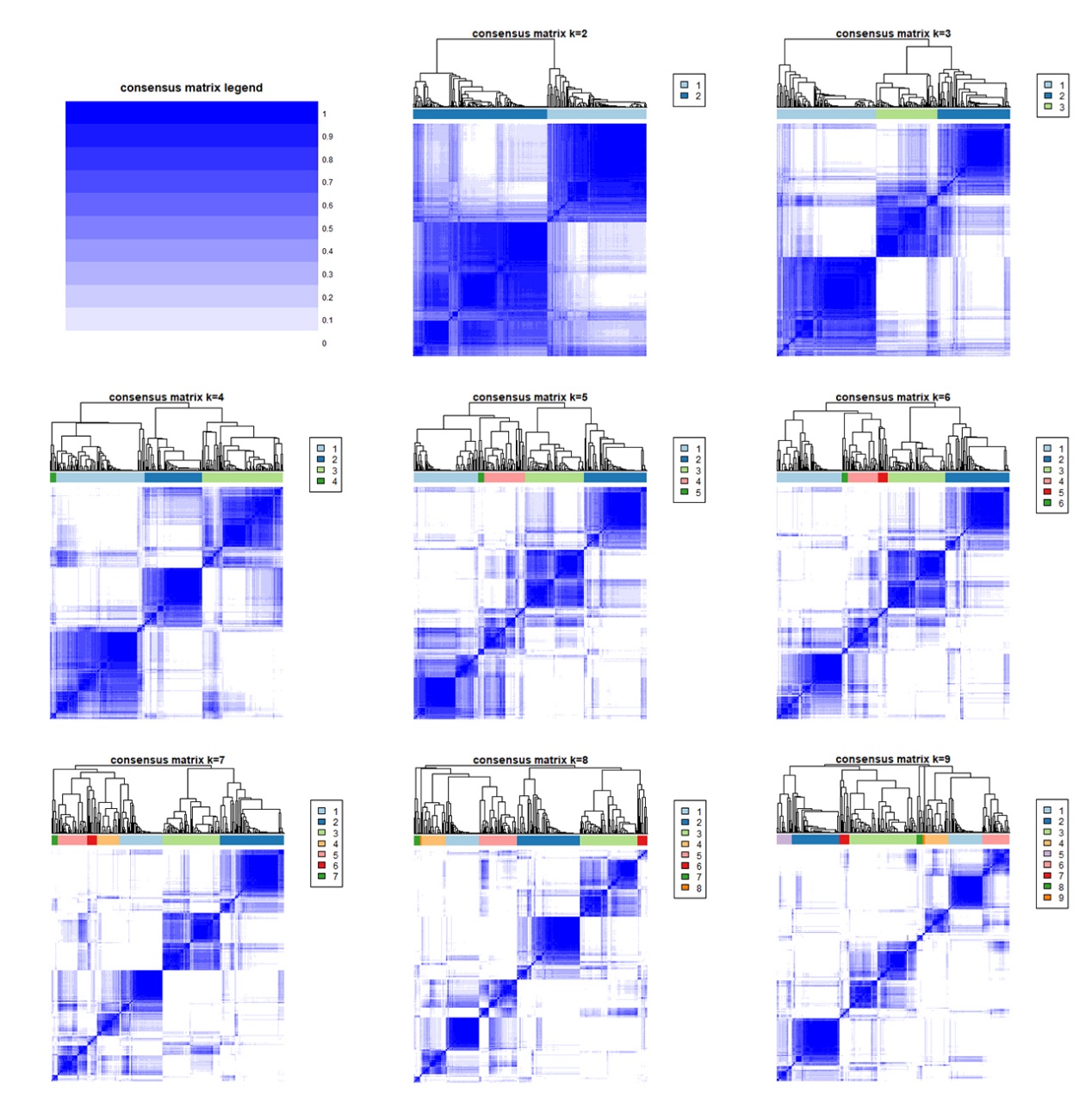


Figure. S1 Consensus clustering matrix from k = 2 to 9 for 9 HBV-related ROS miRNAs


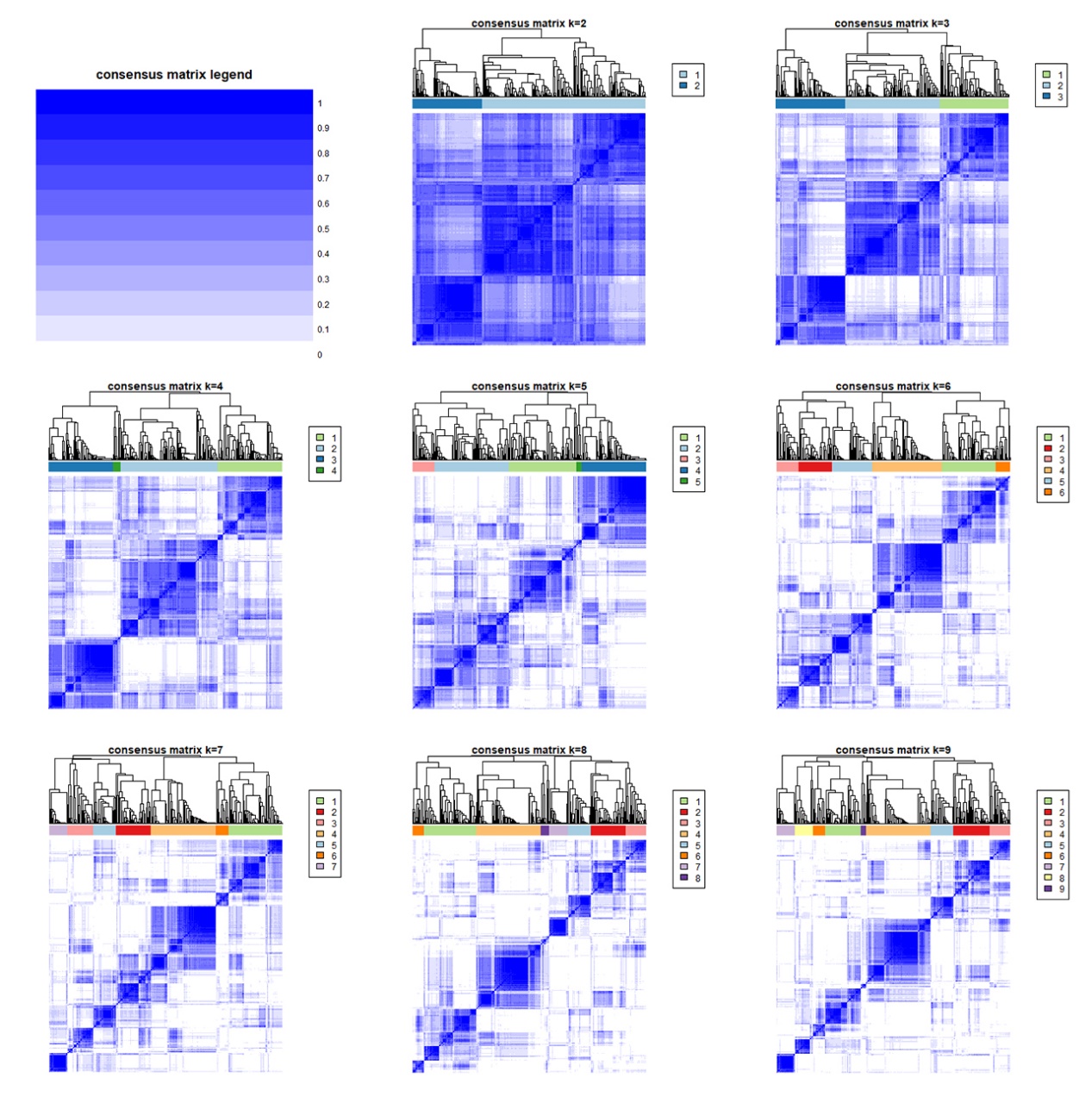


Figure. S2 Consensus clustering matrix from k = 2 to 9 for 27 none HBV-related ROS miRNAs


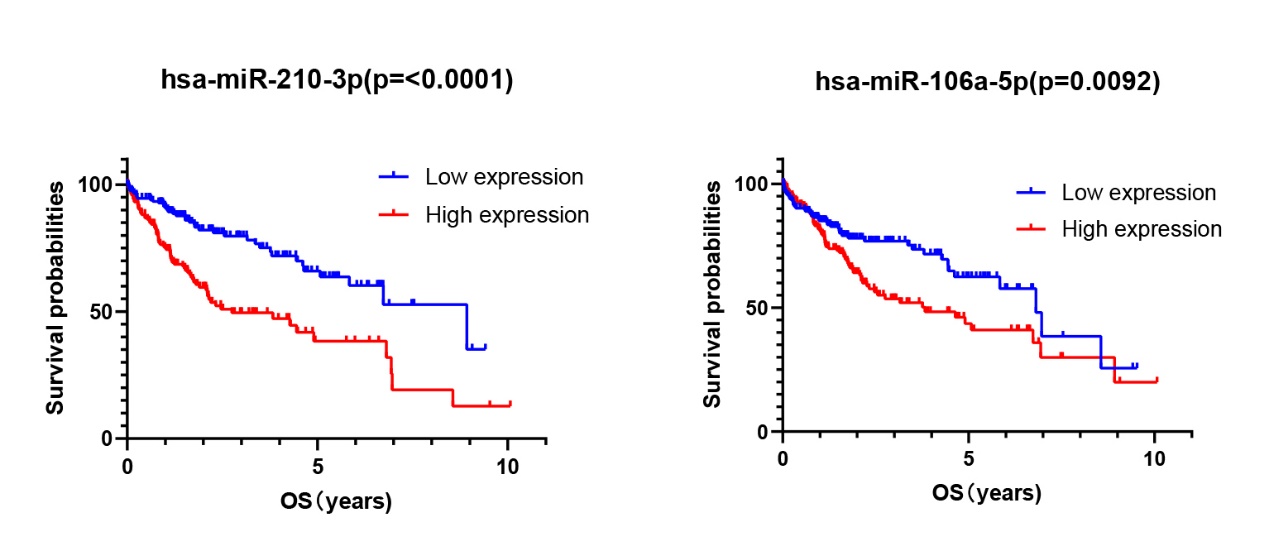


Figure. S3 The OS of miR-210-3p and miR-106a-5p in HCC
